# mRNAs of plants and green algae lack the m^7^G cap‐1 structure

**DOI:** 10.1111/nph.70033

**Published:** 2025-02-27

**Authors:** Chen Xiao, Qiongfang Li, Shangwei Wu, Feng Zhang, Hailei Zhang, Chen Zhang, Zongwei Cai, Yiji Xia

**Affiliations:** ^1^ Department of Biology Hong Kong Baptist University Hong Kong SAR China; ^2^ State Key Laboratory of Environmental and Biological Analysis Hong Kong Baptist University Hong Kong SAR China; ^3^ State Key Laboratory of Agrobiotechnology, School of Life Sciences The Chinese University of Hong Kong Hong Kong SAR China

**Keywords:** Cap‐0, Cap‐1, CapTag‐PAGE, CapTag‐seq, Green algae, m^7^G cap, plant

## Disclaimer

The New Phytologist Foundation remains neutral with regard to jurisdictional claims in maps and in any institutional affiliations.

The 7‐methylguanosine (m^7^G) cap is a characteristic feature found at the 5′ end of eukaryotic mRNA and certain noncoding RNAs. The cap is linked to mRNA through a 5′–5′ pyrophosphate bond (Ramanathan *et al*., 2016). The formation of the cap involves a capping enzyme (CE) that adds the guanosine (G) cap to the 5′ end of a nascent transcript after *c*. 30 nucleotides have been synthesized. This G cap is subsequently methylated by RNA guanosine‐7 methyltransferase (RNMT) to produce the m^7^G cap, which is also referred to as the m^7^G Cap‐0 form (m^7^GpppN; Shuman, 2002; Cowling, 2010). The m^7^G cap plays a vital role in recruiting proteins to form a cap‐binding complex, which is essential for transcription elongation, splicing, polyadenylation, and translation initiation, as well as providing protection from degradation by 5′–3′ exonucleases (Galloway & Cowling, 2019). While m^7^G capping was once considered a constitutive housekeeping process for all eukaryotic mRNA, emerging evidence suggests that it is regulated in a gene‐specific manner in response to various stimuli (Borden *et al*., 2021). Additionally, recent findings indicate that some RNAs in both prokaryotic and eukaryotic organisms possess noncanonical caps, such as the nicotinamide adenine dinucleotide (NAD) cap (Wolfram‐Schauerte & Höfer, 2023), highlighting the complexity of gene regulation mechanisms involving RNA capping.

In addition to the Cap‐0 form, the first transcribed nucleotides of mRNA can be methylated at the 2′ hydroxyl position of its ribose during mRNA biogenesis, resulting in the formation of the Cap‐1 mRNA (m^7^GpppNm) (Galloway & Cowling, 2019). After mRNA is exported into the cytosol, Cap‐1 mRNAs can undergo further methylation at the second nucleotide, also at the 2′ hydroxyl of the ribose, to produce Cap‐2 mRNA (m^7^GpppNmNm).

Mammalian mRNA typically possesses the Cap‐1 structure, whereas mRNAs that possess only the Cap‐0 structure are recognized as nonself RNA, triggering the innate immune response (Daffis *et al*., 2010). Further, a large portion of Cap‐1 mRNAs in human cells is further converted to the Cap‐2 form, whereas an elevated level of Cap‐1 mRNA can still activate the innate immune response although not as potent as Cap‐0 mRNA (Despic & Jaffrey, 2023). Besides preventing excessive activation of the immune response, Cap‐1 and Cap‐2 play additional roles such as mRNA stability and increased translation (Despic & Jaffrey, 2023).

Although it has been known for over 40 yr that plant mRNAs also contain the m^7^G cap (Nichols, 1979; Haugland & Cline, 1980), it remains unclear whether any plant RNAs possess the Cap‐1 or Cap‐2 forms. To further investigate the different forms of mRNA caps and their roles in gene regulation, we aimed to determine whether plant mRNAs also contain Cap‐1 and Cap‐2 structures. Here, we report our findings that mRNAs in both plants and green algae are deficient in the Cap‐1 structure, and consequently, they also do not possess the Cap‐2 structure. This suggests a fundamental evolutionary divergence in the canonical mRNA cap modification and function between animal and plant lineages.

Recently, a method called CapTag‐seq has been developed to quantify and identify Cap‐0, Cap‐1, and Cap‐2 structures in human cells (Despic & Jaffrey, 2023). This method utilizes RNase T2, which cleaves all phosphodiester bonds in RNA except for the bond between 2′‐*O*‐methylated nucleotides and one nucleotide downstream of a 2′‐*O*‐methylated nucleotide (Motorin & Marchand, 2018). In CapTag‐seq, mRNAs are first treated with an m^7^G decapping enzyme to remove 7‐methylguanosine diphosphate (m^7^GDP) from the cap, resulting in a 5′‐monophosphorylated end that is ligated to a 5′ adaptor. The adaptor consists of 2′‐*O*‐methylated nucleotides, making it resistant to RNase T2 cleavage. After ligation to the adaptor, the sample is digested with RNase T2. If the original mRNA contains only the Cap‐0 structure, a single nucleotide (i.e. the first nucleotide) will remain attached to the adaptor, whereas mRNAs with Cap‐1 or Cap‐2 structures will have two and three nucleotides attached to the adaptor, respectively.

**Fig. 1:**
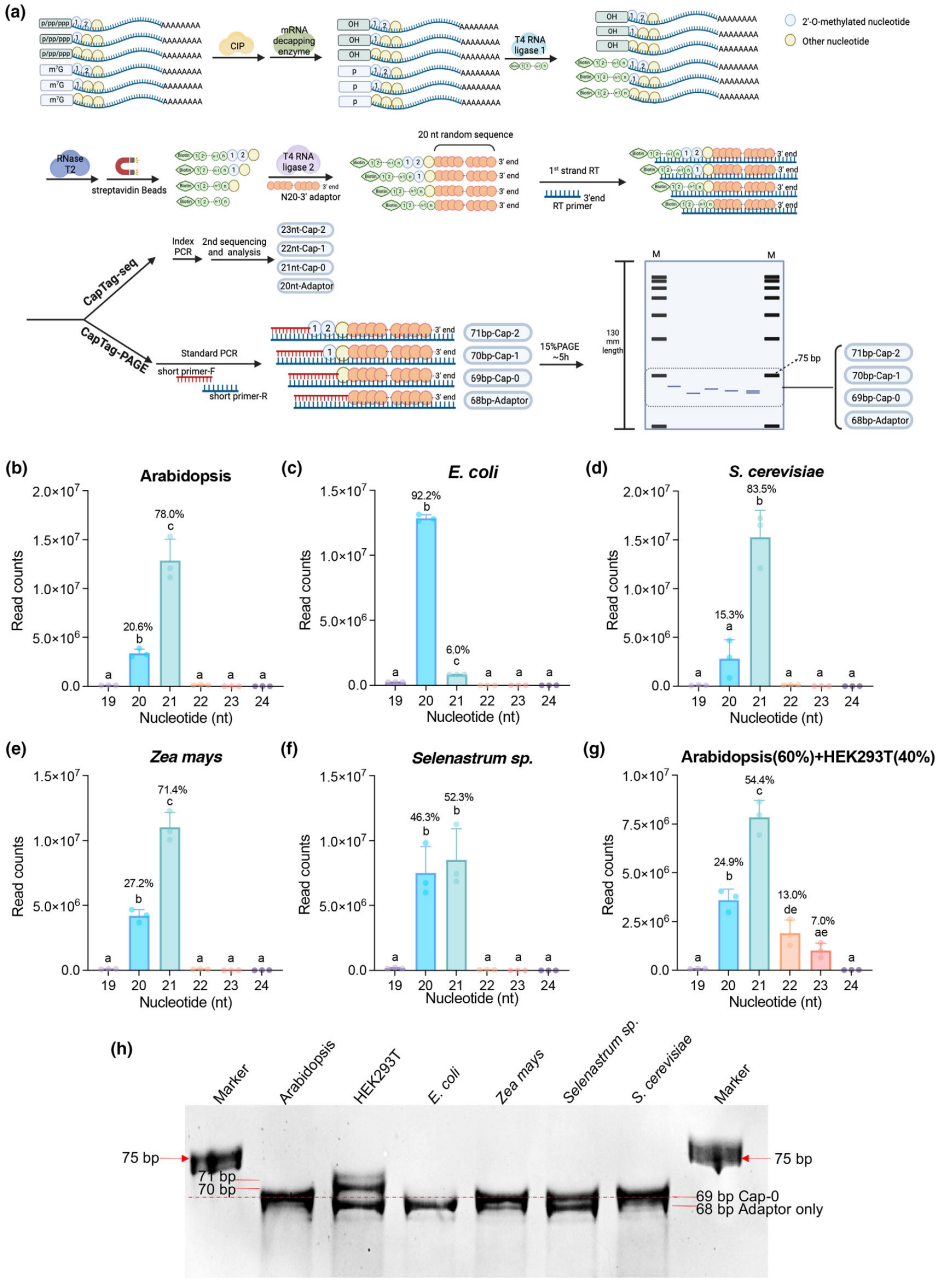
CapTag‐seq and CapTag‐polyacrylamide gel electrophoresis (PAGE) reveal that mRNAs of plants and green algae are deficient in the 7‐methylguanosine Cap‐1 structure. (a) Schematic diagram of the CapTag‐seq and CapTag‐PAGE workflow used in the experiment. The workflow of CapTag‐seq was adopted from (Despic & Jaffrey, 2023). The schematic diagrams were created in BioRender (https://Biorender.com/e74y565). (b–g) Read counts representing different cap structures in mRNAs from Arabidopsis (b), *Escherichia coli* (c), *Saccharomyces cerevisiae* (d), *Zea mays* (e), *Selenastrum* sp. (f), and a mixture of Arabidopsis and HEK293T cells (g). The 20 nt read length indicates self‐ligation of the 5′ and 3′ adapters, while the 21, 22, and 23 nt read lengths correspond to the Cap‐0, Cap‐1, and Cap‐2 structures, respectively. Data represent mean ± SD (*n* = 3), with all individual data points represented as dots. Different letters in (b–e) indicate significant differences as determined by one‐way ANOVA with Tukey's test (*P* < 0.05). (h) Analysis of cap structures using CapTag‐PAGE. This method enables the separation of bands with single‐nucleotide variations in the polymerase chain reaction (PCR) products, ranging from 68 to 71 bp (69 bp for Cap‐0, 70 bp for Cap‐1, and 71 bp for Cap‐2). The red‐dashed line indicates the location of the 69 bp PCR product. CIP, calf intestinal alkaline phosphatase; RT, reverse transcription.

To carry out the CapTag‐seq analysis (Fig. 1a), we extracted total RNAs from Arabidopsis (*Arabidopsis thaliana*), maize (*Zea mays*), green algae (*Selenastrum* sp.), human HEK293T cells, baker's yeast (*Saccharomyces cerevisiae*), and *Escherichia coli* (*E. coli*). Agarose gel electrophoresis indicated that the total RNAs from different organisms were intact (Supporting Information Fig. S1). mRNAs from the eukaryotic organisms were enriched using oligo d(T) beads, while mRNAs from *E. coli* were enriched with an rRNA removal kit.

To minimize the ligation of the 5′ adaptor with uncapped monophosphorylated RNAs that might be present due to partially degraded or sheared RNAs, we first treated the samples with calf intestinal alkaline phosphatase (CIP) to remove the 5′ phosphate. The samples were then subjected to m^7^G decapping, resulting in 5′‐monophosphate RNAs that were ligated to a biotin‐labeled, 2′‐*O*‐methylated 5′ RNA adaptor. Following RNase T2 cleavage, we enriched the RNA fragments containing the adaptor using streptavidin beads. The samples were then ligated to a 3′ adaptor containing a random 20 nt (N20) sequence, and the ligation products were reverse transcribed into complementary DNA (cDNA).

cDNAs were amplified using index polymerase chain reaction (PCR) to create cDNA libraries. We determined that 15 cycles of PCR were optimal for our experimental conditions, yielding sufficient products for sequencing analysis without overamplification. The resulting cDNA libraries were sequenced using Illumina sequencing and analyzed with the SOAPnuke v2.1.9 software (Chen *et al*., 2018). A read of 20 nt indicates a product from self‐ligation of the 5′ and 3′ adaptors, which contains only the N20 sequence, while 21, 22, and 23 nt reads correspond to the Cap‐0, Cap‐1, and Cap‐2 forms, respectively (Fig. 1a).

In the Arabidopsis cDNA library, 78.0% of the total reads were 21 nt reads, indicating their origin from Cap‐0 mRNAs (Fig. 1b). However, these 21 nt reads may not be exclusively derived from Cap‐0 mRNAs, as partially sheared or degraded RNAs might not be completely dephosphorylated and could still ligate to the 5′ adaptor. This observation also explains why 6% of the reads from *E. coli*, which do not possess the Cap‐0 structure, were 21 nt in length, indicating the presence of some 5′‐monophosphate RNAs in the sample during ligation to the 5′ adaptor (Fig. 1c).

In addition to the 21 nt reads in the Arabidopsis library, 20.6% of the reads were 20 nt long, likely resulting from self‐ligation of the two adaptors. The remaining 1.4% of the reads comprised lengths of 19, 22, 23, and 24 nt (Fig. 1b). The minimal presence of 22 and 23 nt reads is unlikely to indicate the presence of Cap‐1 and Cap‐2 mRNAs; rather, it is more likely a result of noise, similar to the minimal levels of 19 and 24 nt reads. This noise could arise from incomplete digestion by RNase T2, sequencing errors, or other factors. This is further supported by the presence of comparable levels of 22 and 23 nt reads in the libraries from *E. coli* and yeast (Fig. 1c,d), which have also been reported to lack Cap‐1 or Cap‐2 structures (Sripati *et al* *.,* 1976). These results suggest that Arabidopsis mRNA does not contain the Cap‐1 or Cap‐2 forms. Similarly, we did not detect Cap‐1 or Cap‐2 mRNAs in maize or green algae (Fig. 1e,f).

The failure to detect Cap‐1 or Cap‐2 structures in the plant and green algae samples is unlikely due to a technical issue, as we have repeated similar experiments multiple times and consistently obtained comparable results. Furthermore, in our experiments, we included an RNA sample as a positive control, in which Arabidopsis mRNAs were mixed with mRNAs from human HEK293T cells at a 60% : 40% ratio. It has been reported that HEK293T cells lack Cap‐0‐only mRNAs (Despic & Jaffrey, 2023); however, their mRNAs generally contain Cap‐1, with a significant portion further methylated to form Cap‐2 (Furuichi *et al*., 1975; Despic & Jaffrey, 2023). In the mixed Arabidopsis–human samples, 54.4% of the reads were 21 nt in length, likely attributed solely to Arabidopsis Cap‐0 mRNAs, while the 22 and 23 nt reads accounted for a total of 20.0% of the total reads (Fig. 1g). These results further support the conclusion that plants and green algae lack the Cap‐1 and Cap‐2 forms.

In addition to the CapTag‐seq method, we employed polyacrylamide gel electrophoresis (PAGE) to analyze the different forms of cap structures. This modified method, which we have named CapTag‐PAGE (Fig. 1a), is more cost‐effective as it does not require sequencing of the cDNA libraries, although it is not as quantitative as CapTag‐seq. In CapTag‐PAGE, RNAs were processed similarly to CapTag‐seq until the synthesis of the first‐strand cDNA via reverse transcription. Subsequently, short primers were added to the cDNA fragments, which were then amplified using standard PCR. The resulting PCR products were separated using 15% PAGE on a long gel (*c*. 130 mm) for *c*. 5 h, allowing for the distinction of products ranging from 68 to 71 bp, representing the origins of adaptor self‐ligation, Cap‐0, Cap‐1, and Cap‐2 mRNAs, with single‐nucleotide resolution. CapTag‐PAGE revealed that mRNAs from plants, green algae, and yeast exclusively contain the m^7^G Cap‐0 structure, as indicated by a clear 69 bp band, with no detectable 70 bp (Cap‐1) or 71 bp (Cap‐2) bands (Fig. 1h). By contrast, only the Cap‐1 and Cap‐2 forms were detectable in human HEK293T cells, as demonstrated by two distinct bands at 70 and 71 bp (Fig. 1h), but no detectable 69 bp (Cap‐0) product. As a negative control, *E. coli* displayed a single clear band at 68 bp, representing the ligation products of the adaptors (Fig. 1h).

From the CapTag‐seq data, we also analyzed the compositions of the first nucleotides in the mRNA samples, which represent the transcription start sites (TSS). All of the organisms exhibited a similar TSS preference, with *c*. 60% being adenine (A) and *c*. 20% being guanine (G), followed by the pyrimidine nucleotide cytosine (C), while thymine (T) was present in the lowest abundance (Fig. S2).

In animals, cap methyltransferase 1 (CMTR1) and cap methyltransferase 2 (CMTR2) catalyze the successive 2′‐*O*‐methylation of the first and second nucleotides, leading to the formation of Cap‐1 and Cap‐2 structures, respectively (Smietanski *et al*., 2014). A homolog search of plant protein sequences in the National Center for Biotechnology Information databases revealed no plant sequences resembling CMTR1 (Fig. S3a). Similarly, a BLAST analysis comparing human CMTR1 with the Arabidopsis TAIR database (Araport11 protein sequences database) also showed no significant matches (Fig. S3b). We cannot entirely dismiss the possibility that a distinct methyltransferase exists in plants, capable of catalyzing the 2′‐*O*‐methylation of the first transcribed nucleotide in mRNAs from certain genes; however, if such a modification is present in plants, it falls below the detection limits of the methods employed in this study.

Although it has been known for over 40 yr that plant mRNAs possess the m^7^G cap, research on the mechanisms of m^7^G capping and its role in gene regulation in plants has been very limited. This may be partly due to the assumption that plants utilize a capping mechanism similar to that of animals. This study reveals that mRNAs in plants and green algae, similar to yeast mRNAs, primarily carry the Cap‐0 structure, with no presence of Cap‐1 or Cap‐2 forms. By contrast, human Cap‐0 mRNAs are generally methylated at the first transcribed nucleotide to form Cap‐1 mRNA, with a significant portion further modified to produce the Cap‐2 structure. Our findings regarding the different forms of mRNA cap structures suggest a fundamental evolutionary divergence in cap modification and function between animals and other eukaryotic organisms, although m^7^G Cap‐0 capping is a common feature that likely evolved in a shared ancestor of these eukaryotic groups. Recently, the CE and RNMT responsible for the formation of the m^7^G cap in Arabidopsis have been identified and characterized, showing significant sequence similarity to these enzymes in animals (Kerk *et al*., 2008; Xiao *et al*., 2023; Ning *et al*., 2024). In addition to these two core enzymes for m^7^G capping, mammalian RNMT is activated by a miniprotein known as RNMT‐activating miniprotein (RAM) (Gonatopoulos‐Pournatzis *et al*., 2011). However, we have recently discovered that Arabidopsis RNMT1 is activated by Decapping and exoribonuclease 1 (DXO1) (Xiao *et al*., 2023), which is also known as a decapping enzyme for NAD‐capped RNAs (Kwasnik *et al*., 2019; Pan *et al*., 2020; Yu *et al*., 2021). By contrast, RAM does not appear to have any other known functions. These findings suggest a unique mechanism in plants for mediating m^7^G capping, potentially linked to NAD capping/decapping. The absence of Cap‐1 and Cap‐2 forms in plants further indicates a distinct mechanism and function of mRNA capping in gene regulation.

## Materials and Methods

### Materials and growth/culture condition

*Arabidopsis thaliana* (L.) Heynh. (Col‐0) was grown on ½ Murashige and Skoog salt medium supplemented with 1% sucrose and 0.8% agar, with the pH adjusted to 5.7 using KOH. *Zea mays* L. (B73) was cultivated in nutrient‐rich soil and watered regularly. *Selenastrum* sp. was cultured in Bristol liquid medium. Plant and green algae samples were placed in a growth room at 16 h : 8 h, 22°C, light : dark photoperiod. *Escherichia coli* (Migula) Castellani & Chalmers (MG1655K12) was cultured in Luria–Bertani liquid medium at 37°C. *Saccharomyces cerevisiae* Meyen ex E.C. Hansen (BY4741) was cultured in yeast extract peptone dextrose liquid medium at 30°C. *Homo sapiens* L. (HEK293T) cells were cultured in Dulbecco's Modified Eagle Medium supplemented with 10% fetal bovine serum at 37°C in a 5% CO_2_ atmosphere.

### Oligonucleotide primers

The primer sequences used in this study are listed in Table S1.

### Total RNA extraction

Plant and green algae samples were ground to a powder in liquid nitrogen using a mortar and pestle. For *Selenastrum* sp., polyvinylpyrrolidone was added for fully grinding. Twelve‐day‐old Arabidopsis seedlings and *Z. mays* leaves were harvested for total RNA extraction by using PureLink™ Plant RNA Reagent (Thermo Fisher) according to the manufacturer's instructions. Briefly, 2 g of samples was incubated with 10 ml of the RNA reagent at room temperature and then centrifuged. The supernatant was thoroughly mixed with one‐fifth volume of 5 M NaCl, followed by the addition of three‐fifth volume of chloroform. After centrifugation, the upper aqueous phase containing RNA was transferred into a new tube, and the RNA was eluted with ethanol.

Both *Selenastrum* sp. and HEK293T cells were extracted using Trizol, with 1 ml Trizol used for every 10^6^ cells. After Trizol incubation, the RNAs were mixed with one‐fifth volume chloroform and eluted with ethanol.

For *S. cerevisiae* and *E. coli*, cells were collected when the OD_600_ reached to 0.6. For every 1 ml cells, 60 μl Tris–EDTA–SDS buffer and 40 μl acid phenol: chloroform, pH 4.5 (Thermo Fisher, Waltham, MA, USA), were added. The cell pellet was resuspended in this mixture and incubated at 65°C for 15 min. RNA was then extracted with acid phenol: chloroform and chloroform, followed by ethanol elution.

After ethanol elution and washing steps, the RNA precipitates were dissolved in RNase‐free water and incubated with DNase I (NEB, Ipswich, MA, USA) to remove any DNA residue. The RNA was then extracted with chloroform, eluted with ethanol, and finally dissolved in RNase‐free water.

### mRNA enrichment

mRNA from *E. coli* was enriched using the MICROBExpress™ Bacterial mRNA Enrichment Kit (Invitrogen, Waltham, MA, USA) according to the manufacturer's instructions. This method captures rRNA with oligo magnetic beads and recovers mRNA for enrichment. For other species in this study, mRNA was enriched in two rounds with Oligo d(T)25 magnetic beads (NEB). Specifically, mRNA was bound with Oligo d(T)25 beads in 1X RNA binding buffer (1 M LiCl, 40 mM Tris‐HCl with pH 7.5, 2 mM Ethylenediaminetetraacetic acid (EDTA), and 0.1% Triton X‐100) at room temperature for 30 min, followed by two washes with RNA wash buffer (150 mM LiCl, 20 mM Tris‐HCl with pH 7.5, 1 mM EDTA, and 0.01% Triton X‐100). The bound mRNA was eluted with 10 mM Tris‐HCl (pH 7.5) at 80°C for 2 min, allowed to cool to room temperature, and then mixed with binding buffer to enable rebinding to the beads for a second‐round enrichment. After washing, the mRNA was finally re‐eluted with Tris‐HCl buffer.

The concentration of enriched mRNA was measured using a Qubit Fluorometer with the RNA HS Assay kit (Thermo Fisher).

### CapTag‐seq library preparation

The CapTag‐seq library preparation was conducted according to a previously reported procedure with minor modification (Despic & Jaffrey, 2023). Two microgram mRNA was treated with 25 U Quick CIP (NEB) to remove phosphate groups from the 5′ end of uncapped RNA, which blocks ligation between the 5′ adaptor and uncapped RNA. This was followed by treatment with 1 μl mRNA decapping enzyme (NEB) in a 20 μl reaction, converting the m^7^G capped RNA into monophosphate RNA. The monophosphate RNA product was then ligated to 30 pmol biotin‐labeled 2′‐*O*‐methylated 5′ adaptors (IDT, Coralville, IA, USA) using 50 U T4 RNA ligase 1 (NEB) at 25°C for 4 h in a 30 μl reaction. RNaseT2 (Worthington Biochemical, Worthington, OH, USA) was dissolved in a solution of 15 mM NaOAc, 100 mM NaCl, and 50% glycerol. RNase T2 treatment was conducted in 30 mM NaOAc (pH 4.5) at 37°C for *c*. 14 h. The resulting biotin‐adaptor ligated cap nucleotides (ranging from mononucleotide to trinucleotide) were captured with Dynabeads™ MyOne™ Streptavidin T1 beads (Invitrogen). 10 U T4 polynucleotide kinase (NEB) was used to remove 3′‐phosphoryl groups from the 3′ end of the cap nucleotides. The bead‐bound RNA product was then ligated to a 3′ adaptor linked to a pre‐adenylated random 20 nt (N20) sequence at 27°C for 4 h. Nonligated N20‐3′ adaptors were further removed with 25 U yeast 5′‐deadenylase (NEB) and 15 U RecJf (NEB) at 30°C for 45 min. The RNA cap nucleotide, now ligated with the 5′ adaptor and N20‐3′ adaptor, was eluted from beads with biotin elution buffer (10 mM Tris‐HCl, 50 mM NaCl, 1 mM EDTA, and 1 mM D‐biotin) at 37°C for 30 min. To remove D‐biotin from the RNA product, a Zymo RCC‐5 column was used according to the manufacturer's instruction for isolating RNA longer than 17 nt. The RNA was then first‐strand transcribed into cDNA with RT primer using SuperScript III (Invitrogen), followed by 15 cycle index PCR with Q5 High‐Fidelity DNA Polymerase (NEB). The product was purified with 1.8× PCR volume of NEBNext sample purification beads (NEB) and eluted with 20 μl 0.1× Tris‐EDTA (TE) buffer. cDNA libraries were sent to Novogene Co. (Beijing, China) for 50‐bp single‐end Illumina sequencing.

### CapTag‐seq data analysis

Raw reads were filtered and trimmed, and duplicate reads were removed using SOAPnuke v2.1.9 (Chen *et al*., 2018). The count of clean reads ranging from 19 to 24 nt and the count of the first nucleotide in 21 nt reads were recorded.

### CapTag‐PAGE

The first‐strand cDNA, generated in the same way as during CapTag‐seq library preparation, was amplified using standard PCR with two short primers (PCR short primer‐F and PCR short primer‐R) and Q5 High‐Fidelity DNA Polymerase (NEB). Specifically, this reaction was carried out in a 10 μl mixture containing 2.5 μl cDNA, 2 μl 5× Q5 reaction buffer, 0.2 μl 10 mM Deoxyribonucleotide triphosphate (dNTP), 0.4 μl 10 μM PCR short primer‐F and R, and 0.2 U Q5 DNA polymerase. The PCR products, ranging from 68 to 71 bp, were analyzed with 15% native PAGE in 1× TBE buffer at 300 V for 5 h. A low‐molecular‐weight DNA ladder (NEB) was used in this assay.

## Competing interests

None declared.

## Author contributions

YX, CX and ZC planned and designed the research plan. CX, SW, FZ, HZ and CZ performed the experiments. QL and CX did bioinformatics analysis of the CapTag‐seq data. All authors contributed to data analysis and manuscript writing.

## Supporting information


**Fig. S1** Analysis of total RNA integrity.
**Fig. S2** Compositions of the first nucleotides in mRNAs from Arabidopsis, Arabidopsis+HEK293T, *Zea mays*, *Selenastrum* sp., and *Saccharomyces cerevisiae*.
**Fig. S3** Blast results of human cap methyltransferase1.
**Table S1** Primers used in this study.
Please note: Wiley is not responsible for the content or functionality of any Supporting Information supplied by the authors. Any queries (other than missing material) should be directed to the *New Phytologist* Central Office.

## Data Availability

Data are provided in the article and Supporting Information. The CapTag‐seq data have been submitted to the Sequence Read Archive of the National Center for Biotechnology Information, and the BioProject ID is PRJNA1200270.
