# Supplementary material for: mRNAs of plants and green algae lack the m7G cap‐1 structure
Source: New Phytol. 2025 Feb 27;246(2):396–401. doi: 10.1111/nph.70033 (PMC11923408; doi:10.1111/nph.70033)
Supplement: Supplementary file 1 — Fig. S1 Analysis of total RNA integrity. Fig. S2 Compositions of the first nucleotides in mRNAs from Arabidopsis, Arabidopsis+HEK293T, Zea mays, Selenastrum sp., and Saccharomyces cerevisiae. Fig. S3 Blast results of human cap methyltransferase1. Table S1 Primers used in this study. Please note: Wiley is not responsible for the content or functionality of any Supporting Information supplied by the authors. Any queries (other than missing material) should be directed to the New Phytologist Central Office. [file NPH-246-396-s001.pdf]

New Phytologist

***New Phytologist* Supporting Information**

Article title: mRNAs of Plants and Green Algae Lack the m^7G Cap-1 Structure

Authors: Chen Xiao1, Qiongfang Li1, Shangwei Wu1, Feng Zhang2, Hailei Zhang1, Chen Zhang1, Zongwei Cai2, Yiji Xia1,2,3

Article acceptance date: 05 February 2025

The following Supporting Information is available for this article:

Fig. S1 Analysis of total RNA integrity.

Fig. S2 The compositions of the first nucleotides in mRNAs from Arabidopsis,

Arabidopsis+HEK293T, *Zea mays*, *Selenastrum sp.* and *S. cerevisiae*.

Fig. S3 BLAST results of human cap methyltransferase1 (CMTR1).

Table S1 Primers used in this study

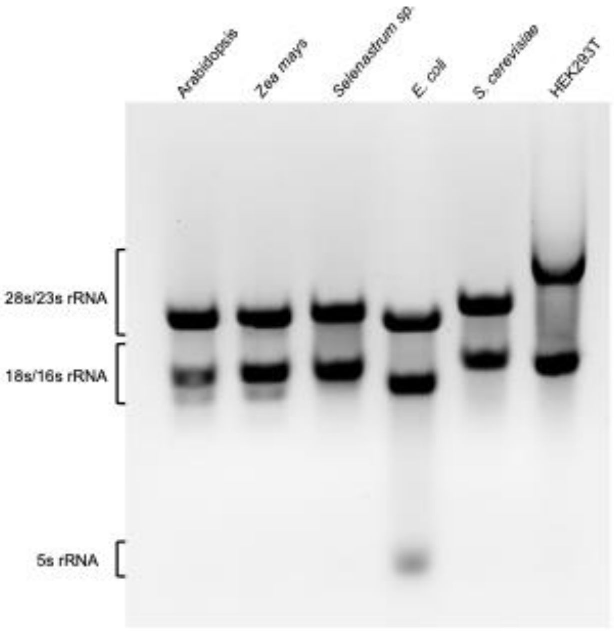

Fig. S1 Analysis of total RNA integrity. Total RNA was extracted using PureLink™ Plant RNA Reagent (for Arabidopsis and *Zea mays*), Trizol Reagent (for *Selenastrum sp.* and HEK293T), and SDS and acid-phenol: chloroform (for *S. cerevisiae* and *E. coli*). To access the RNA quality, 1.5μg total RNA samples were analyzed by running 1% agarose gel electrophoresis.

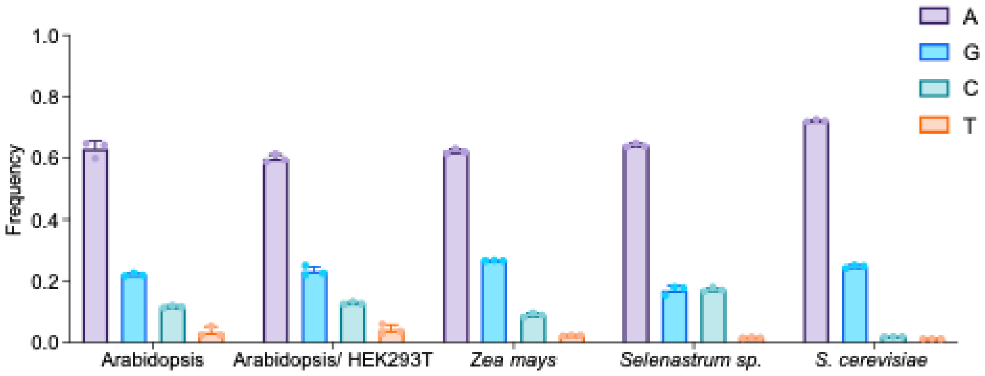

Fig. S2 The compositions of the first nucleotides in mRNAs from Arabidopsis, Arabidopsis+HEK293T, *Zea mays*, *Selenastrum sp.* and *S. cerevisiae*. The Y-axis represents the nucleotide frequency at the transcription start site. Data represent mean ± SD (n = 3), with all individual data points represented as dots.

(**a**)

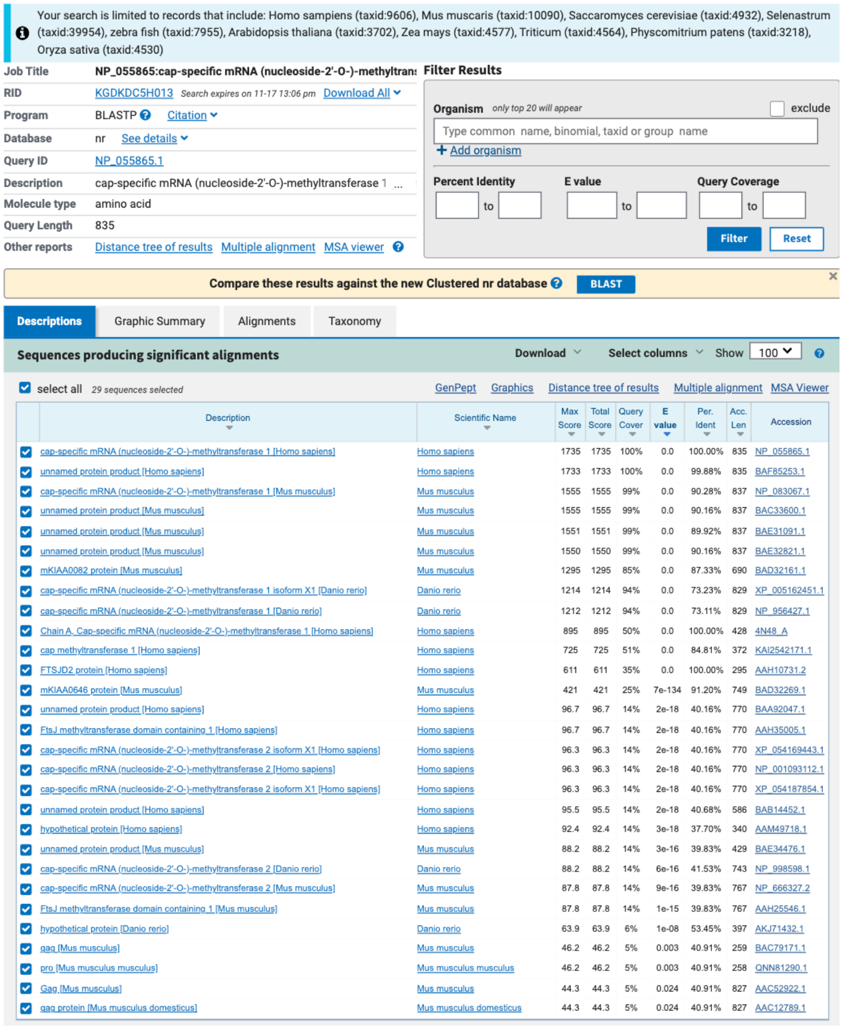

Description	Scientific Name	Max Score	Total Score	Query Cover	E value	Per. Ident	Acc. Len	Accession
cap-specific mRNA (nucleoside-2'-O-)-methyltransferase 1 [Homo sapiens]	Homo sapiens	1735	1735	100%	0.0	100.00%	835	NP_055865.1
unnamed protein product [Homo sapiens]	Homo sapiens	1733	1733	100%	0.0	99.88%	835	BAF85253.1
cap-specific mRNA (nucleoside-2'-O-)-methyltransferase 1 [Mus musculus]	Mus musculus	1555	1555	99%	0.0	90.28%	837	NP_083067.1
unnamed protein product [Mus musculus]	Mus musculus	1555	1555	99%	0.0	90.16%	837	BAC33600.1
unnamed protein product [Mus musculus]	Mus musculus	1551	1551	99%	0.0	89.92%	837	BAE31091.1
unnamed protein product [Mus musculus]	Mus musculus	1550	1550	99%	0.0	90.16%	837	BAE32821.1
mKIAA0082 protein [Mus musculus]	Mus musculus	1295	1295	85%	0.0	87.33%	690	BAD32161.1
cap-specific mRNA (nucleoside-2'-O-)-methyltransferase 1 isoform X1 [Danio rerio]	Danio rerio	1214	1214	94%	0.0	73.23%	829	XP_005162451.1
cap-specific mRNA (nucleoside-2'-O-)-methyltransferase 1 [Danio rerio]	Danio rerio	1212	1212	94%	0.0	73.11%	829	NP_956427.1
Chain A, Cap-specific mRNA (nucleoside-2'-O-)-methyltransferase 1 [Homo sapiens]	Homo sapiens	895	895	50%	0.0	100.00%	428	4N48_A
cap methyltransferase 1 [Homo sapiens]	Homo sapiens	725	725	51%	0.0	84.81%	372	KAI2542171.1
FTSJD2 protein [Homo sapiens]	Homo sapiens	611	611	35%	0.0	100.00%	295	AAH10731.2
mKIAA0646 protein [Mus musculus]	Mus musculus	421	421	25%	7e-134	91.20%	749	BAD32269.1
unnamed protein product [Homo sapiens]	Homo sapiens	96.7	96.7	14%	2e-18	40.16%	770	BAA92047.1
FtsJ methyltransferase domain containing 1 [Homo sapiens]	Homo sapiens	96.7	96.7	14%	2e-18	40.16%	770	AAH35005.1
cap-specific mRNA (nucleoside-2'-O-)-methyltransferase 2 isoform X1 [Homo sapiens]	Homo sapiens	96.3	96.3	14%	2e-18	40.16%	770	XP_054169443.1
cap-specific mRNA (nucleoside-2'-O-)-methyltransferase 2 [Homo sapiens]	Homo sapiens	96.3	96.3	14%	2e-18	40.16%	770	NP_001093112.1
cap-specific mRNA (nucleoside-2'-O-)-methyltransferase 2 isoform X1 [Homo sapiens]	Homo sapiens	96.3	96.3	14%	2e-18	40.16%	770	XP_054187854.1
unnamed protein product [Homo sapiens]	Homo sapiens	95.5	95.5	14%	2e-18	40.68%	586	BAB14452.1
hypothetical protein [Homo sapiens]	Homo sapiens	92.4	92.4	14%	3e-18	37.70%	340	AAM49718.1
unnamed protein product [Mus musculus]	Mus musculus	88.2	88.2	14%	3e-16	39.83%	429	BAE34476.1
cap-specific mRNA (nucleoside-2'-O-)-methyltransferase 2 [Danio rerio]	Danio rerio	88.2	88.2	14%	6e-16	41.53%	743	NP_998598.1
cap-specific mRNA (nucleoside-2'-O-)-methyltransferase 2 [Mus musculus]	Mus musculus	87.8	87.8	14%	9e-16	39.83%	767	NP_666327.2
FtsJ methyltransferase domain containing 1 [Mus musculus]	Mus musculus	87.8	87.8	14%	1e-15	39.83%	767	AAH25546.1
hypothetical protein [Danio rerio]	Danio rerio	63.9	63.9	6%	1e-08	53.45%	397	AKJ71432.1
gag [Mus musculus]	Mus musculus	46.2	46.2	5%	0.003	40.91%	259	BAC79171.1
pro [Mus musculus musculus]	Mus musculus musculus	46.2	46.2	5%	0.003	40.91%	258	QNN81290.1
Gag [Mus musculus]	Mus musculus	44.3	44.3	5%	0.024	40.91%	827	AAC52922.1
gag protein [Mus musculus domesticus]	Mus musculus domesticus	44.3	44.3	5%	0.024	40.91%	827	AAC12789.1

(**b**)

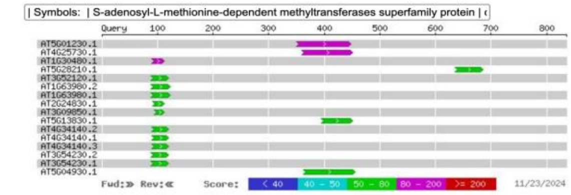


```
Length=835
                                                                    Score      E
Sequences producing significant alignments:                        (Bits)   Value

AT5G01230.1 | Symbols: | S-adenosyl-L-methionine-dependent methyl...  44.7     2e-04
AT4G25730.1 | Symbols: | FtsJ-like methyltransferase family prote...  41.6     0.002
AT1G30480.1 | Symbols: DRT111 | D111/G-patch domain-containing pr...  36.2     0.097
AT5G28210.1 | Symbols: | mRNA capping enzyme family protein | chr...  34.3     0.42
AT3G52120.1 | Symbols: | SWAP (Suppressor-of-White-APricot)/surp ...  33.9     0.44
AT1G63980.2 | Symbols: | D111/G-patch domain-containing protein |...  33.9     0.53
AT1G63980.1 | Symbols: | D111/G-patch domain-containing protein |...  33.9     0.53
AT2G24830.1 | Symbols: | zinc finger (CCCH-type) family protein /...  33.1     0.98
AT3G09850.1 | Symbols: | D111/G-patch domain-containing protein |...  32.7     1.3
AT5G13830.1 | Symbols: | FtsJ-like methyltransferase family prote...  31.2     2.2
AT4G34140.2 | Symbols: | D111/G-patch domain-containing protein |...  31.2     3.7
AT4G34140.1 | Symbols: | D111/G-patch domain-containing protein |...  31.2     3.7
AT4G34140.3 | Symbols: | D111/G-patch domain-containing protein |...  31.2     3.8
AT3G54230.2 | Symbols: SUA | suppressor of abi3-5 | chr3:20073872...  30.8     4.9
AT3G54230.1 | Symbols: SUA | suppressor of abi3-5 | chr3:20073872...  30.8     5.0
AT5G04930.1 | Symbols: ALA1 | aminophospholipid ATPase 1 | chr5:1...  30.4     6.4
```

Fig. S3 BLAST results of human cap methyltransferase1 (CMTR1). (a) Protein sequence alignment was performed using the National Center for Biotechnology Information (NCBI) database to identify homologous genes of CMTR1 in *Homo sapiens*, *Mus musculus*, *Saccharomyces cerevisiae*,

Selenastrum, *Danio rerio* (zebrafish), *Arabidopsis thaliana*, *Zea mays*, *Triticum*, *Physcomitrium patens*, and *Oryza sativa*. (b) Blast results of CMTR1 obtained from TAIR website by using Araport11 protein sequences dataset.

Table S1 Primers used in this study

Name	Sequence (5'-3')	Application
5' adapter	/5BiodT/mGmUmUmCmAmGmAmGmUmUmCmUm AmCmAmGmUmCmCmxGmAmCmGmAmUmC	5' ligation
N_{20}-3' adapter	/5rApp/NNNNNNNNNNNNNNNNNNNNTGGAATTC TCGGGTGCCAAGG/3ddC/	3' ligation
RT primer/ PCR short primer-R	GCCTTGGCACCCGAGAATTCCA	first-strand cDNA generation and library validation
Universal PCR primer	AATGATACGGCGACCACCGAGATCTACACGTTCA GAGTTCTACAGTCCGA	PCR in library preparation
PCR primer index 1	CAAGCAGAAGACGGCATACGAGAT[CGTGAT]GT GACTGGAGTTCCTTGGCACCCGAGAATTCCA	PCR in library preparation
PCR primer index 2	CAAGCAGAAGACGGCATACGAGAT[ACATCG]GT GACTGGAGTTCCTTGGCACCCGAGAATTCCA	PCR in library preparation
PCR primer index 3	CAAGCAGAAGACGGCATACGAGAT[GCCTAA]GT GACTGGAGTTCCTTGGCACCCGAGAATTCCA	PCR in library preparation
PCR primer index 4	CAAGCAGAAGACGGCATACGAGAT[TGGTCA]GT GACTGGAGTTCCTTGGCACCCGAGAATTCCA	PCR in library preparation
PCR primer index 5	CAAGCAGAAGACGGCATACGAGAT[CACTGT]GT GACTGGAGTTCCTTGGCACCCGAGAATTCCA	PCR in library preparation
PCR primer index 6	CAAGCAGAAGACGGCATACGAGAT[ATTGGC]GT GACTGGAGTTCCTTGGCACCCGAGAATTCCA	PCR in library preparation
PCR primer index 7	CAAGCAGAAGACGGCATACGAGAT[GATCTG]GT GACTGGAGTTCCTTGGCACCCGAGAATTCCA	PCR in library preparation
PCR primer index 8	CAAGCAGAAGACGGCATACGAGAT[TCAAGT]GT GACTGGAGTTCCTTGGCACCCGAGAATTCCA	PCR in library preparation
PCR primer index 9	CAAGCAGAAGACGGCATACGAGAT[CTGATC]GT GACTGGAGTTCCTTGGCACCCGAGAATTCCA	PCR in library preparation
PCR primer index 10	CAAGCAGAAGACGGCATACGAGAT[AAGCTA]GT GACTGGAGTTCCTTGGCACCCGAGAATTCCA	PCR in library preparation
PCR primer index 12	CAAGCAGAAGACGGCATACGAGAT[TACAAG]GT GACTGGAGTTCCTTGGCACCCGAGAATTCCA	PCR in library preparation
PCR primer index 13	CAAGCAGAAGACGGCATACGAGAT[TTGACT]GTG ACTGGAGTTCCTTGGCACCCGAGAATTCCA	PCR in library preparation
PCR short primer-F	GTTCAGAGTTCTACAGTCCGA	library validation
